# Ultra-low threshold polariton lasing at room temperature in a GaN membrane microcavity with a zero-dimensional trap

**DOI:** 10.1038/s41598-017-06125-y

**Published:** 2017-07-17

**Authors:** R. Jayaprakash, F. G. Kalaitzakis, G. Christmann, K. Tsagaraki, M. Hocevar, B. Gayral, E. Monroy, N. T. Pelekanos

**Affiliations:** 1 Department of Materials Science and Technology, University of Crete, P.O. Box 2208, 71003 Heraklion, Greece; 2 Microelectronics Research Group, IESL-FORTH, P.O. Box 1385, 71110 Heraklion, Greece; 3 Université Grenoble-Alpes, 38000 Grenoble, France; 4 CNRS, Inst NEEL, F-38000 Grenoble, France; 5 CEA, INAC-PHELIQS, 17 rue des Martyrs, 38000 Grenoble, France

## Abstract

Polariton lasers are coherent light sources based on the condensation of exciton-polaritons in semiconductor microcavities, which occurs either in the kinetic or thermodynamic (Bose-Einstein) regime. Besides their fundamental interest, polariton lasers have the potential of extremely low operating thresholds. Here, we demonstrate ultra-low threshold polariton lasing at room temperature, using an all-dielectric, GaN membrane-based microcavity, with a spontaneously-formed zero-dimensional trap. The microcavity is fabricated using an innovative method, which involves photo-electrochemical etching of an InGaN sacrificial layer and allows for the incorporation of optimally-grown GaN active quantum wells inside a cavity with atomically-smooth surfaces. The resulting structure presents near-theoretical Q-factors and pronounced strong-coupling effects, with a record-high Rabi splitting of 64 meV at room-temperature. Polariton lasing is observed at threshold carrier densities 2.5 orders of magnitude lower than the exciton saturation density. Above threshold, angle-resolved emission spectra reveal an ordered pattern in k-space, attributed to polariton condensation at discrete levels of a single confinement site. This confinement mechanism along with the high material and optical quality of the microcavity, accounts for the enhanced performance of our polariton laser, and pave the way for further developments in the area of robust room temperature polaritonic devices.

## Introduction

Exciton-polaritons are composite half-photon half-exciton states, existing inside semiconductor microcavities in the so called “strong-coupling” regime^1^. Due to their bosonic nature, polaritons were identified early-on, as a means to produce an unconventional “laser”, which does not require population inversion^2^. From a device point of view, polariton lasers are attractive for their potential to display extremely low thresholds of operation in comparison with conventional semiconductor lasers^3^. Room temperature operation of a GaAs polariton light emitting diode has been previously demonstrated^4, 5^. However, polariton lasers using GaAs^6–9^ or CdTe^10^ active layers are limited to cryogenic temperatures, due to their relatively weak exciton binding energy. Wide bandgap semiconductors are better suited for high temperature operation, based on their higher exciton binding energy and oscillator strength, as well as more efficient carrier relaxation due to enhanced exciton-phonon interaction. Several polariton lasing experiments at 300 K have been reported using GaN^11–14^ or ZnO^15, 16^ as active layers. GaN is the most interesting of these materials in view of its capabilities for electrical injection, since it can be doped both n- and p-type.

Previous demonstrations of GaN polariton lasing relied on bulk material^11^, quantum wells (QWs)^12^ or even nanowires^13^ as active medium. Realizations using bulk GaN or QWs present limitations imposed by the modest optical quality of the active layers, grown on highly mismatched silicon substrates^17^, or on strongly disordered AlInN/AlGaN distributed Bragg reflectors (DBRs)^18^. Better performance (lower threshold) is achieved in the case of a single defect-free GaN nanowire embedded in an all-dielectric microcavity. However, the nanowire approach is hardly scalable and electrical injection schemes are difficult to implement. Recently, an electrically injected polariton laser has been demonstrated, using bulk GaN as active layer in an in-plane edge-emitting microcavity^14^. The device has shown low current thresholds, but is rather complex to fabricate and certainly defies the benefits of vertical emission geometry.

In this article, we produce an ultra-low threshold, optically-pumped GaN QW polariton laser in the standard vertical geometry, operating at room temperature. Unlike previous works, the laser is fabricated by utilizing a photo-electrochemical (PEC) etching method^19–24^, which makes it possible to use *optimally-grown* GaN/AlGaN QWs as active medium, embedded in a 3λ/2-thick membrane with *atomically-smooth* surfaces. The PEC method is completed by the deposition of dielectric DBRs on both sides of the membrane. It should be noted that the method is fully compatible with electrical injection schemes, provided an appropriate PIN structure is incorporated within the membranes and contacts are made directly on them to bypass the high resistivity of the dielectric DBRs. The strong coupling regime is observed at 300 K with a Rabi splitting as high as 64 meV. Polariton lasing is observed under quasi-continuous excitation at an average threshold pumping power P_th_ = 4.5 W/cm^2^, corresponding to a threshold carrier density 2.5 orders of magnitude lower than the exciton saturation density. Such threshold is the lowest reported so far for a bulk or QW GaN polariton laser system. Interestingly, angle-resolved photoluminescence (PL) experiments above threshold, exhibit an ordered condensation pattern in k-space, attributed to quantum confinement of polaritons at a single trapping site. We argue that this trapping mechanism along with the high material and optical quality of the microcavity are at the origin of the ultra-low threshold observed in our polariton laser.

## Results

**Figure 1:**
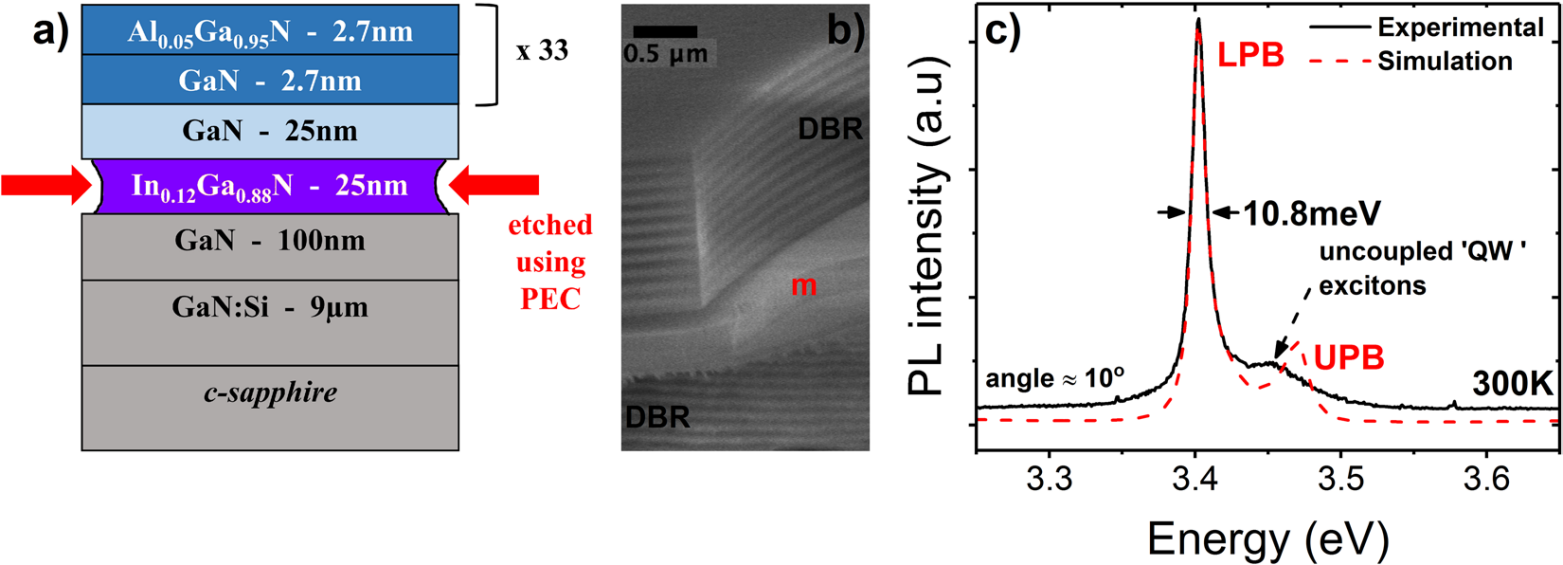
Fabrication of an all-dielectric microcavity. (**a**) Basic structure of the sample under study, highlighting the InGaN sacrificial layer to be removed by photo-electrochemical etching. (**b**) SEM image of a full microcavity, where we distinguish the 3λ/2-thick membrane (“m”) sandwiched between two dielectric DBR mirrors. (**c**) PL spectrum at an incident angle of 10° (black line), from a full microcavity sample, along with its simulation (dashed red line). The LPB and the uncoupled ‘QW’ exciton lines are visible in the spectrum. The expected UPB location is indicated.

The main steps for the fabrication of our GaN microcavity are illustrated in Figs 1 and S1, with details given in Methods. A schematic of the sample structure is shown in Fig. 1a, including the sacrificial InGaN layer to be removed by PEC etching. The sample is grown by molecular beam epitaxy on a GaN/Al_2_O_3_ template, in the optimal growth conditions to obtain a high optical quality of the active region. The quality of the as-grown GaN/Al_0.05_Ga_0.95_N QWs is confirmed prior to etching, by optical characterization. Specifically, the QW PL linewidth measured at 20 K is merely 7 meV, which is a state of the art value for GaN/AlGaN QWs^25^, going up to 28 meV at 300 K. The PL intensity drops only by a factor of 24 between 20 K and 300 K, whereas the lifetime of the QW emission at room temperature remains quite long, around 275 ps (Supplementary Fig. S2). The latter reflects the fact that non-radiative recombination channels are significantly reduced in our structure, compared to the QWs grown on monolithic nitride DBR where the room temperature lifetime is 10 ps^26^. The above characteristics make these optimally-grown QWs excellent candidates for polaritonic devices. Following PEC etching, the etched surface of the detached membranes is atomically smooth, as confirmed by atomic force microscopy (AFM) returning a root-mean-square (RMS) roughness of barely 0.65 nm over a 5 × 5 μm^2^ of scanned area (Supplementary Fig. S1). This value should be compared with the 3.4 nm RMS roughness^26^, measured on the surface of a monolithic AlInN/GaN DBR used in hybrid GaN polariton lasers^11, 12^. In the next step, the membranes are transferred onto a bottom SiO_2_/Ta_2_O_5_ DBR, thus forming a half-microcavity. The reflectivity spectrum from such a half-microcavity at 300 K is shown in Supplementary Fig. S1c, depicting a pronounced negatively-detuned cavity mode with a Q-factor of ~85, along with a QW excitonic feature. A prior knowledge of the cavity mode position is crucial for the precise design and deposition of the top HfO_2_/Al_2_O_3_ DBR in order to form a full microcavity, as shown in Fig. 1b.

**Figure 2:**
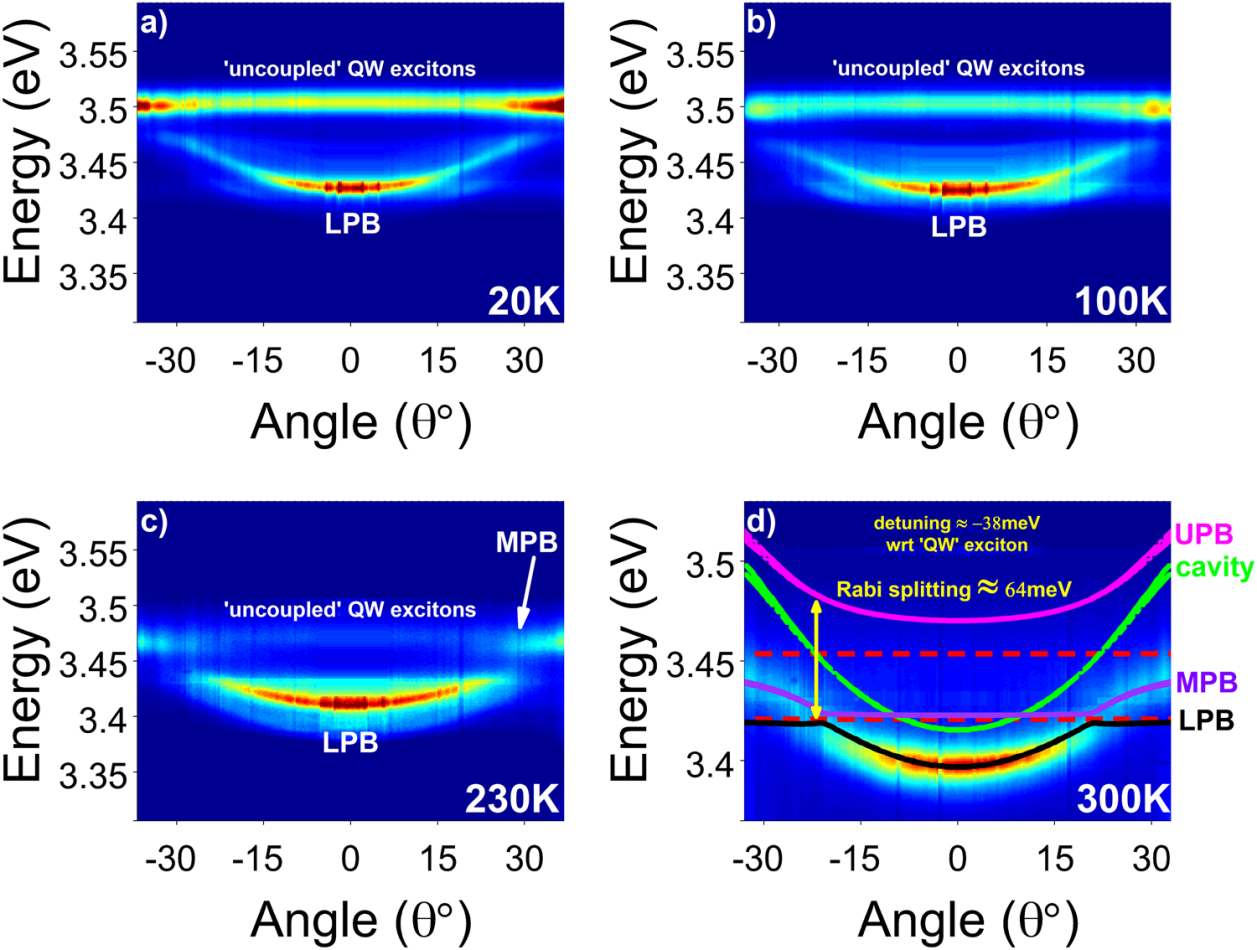
Angle-dependent polariton photoluminescence recorded at various temperatures. (**a**) 20 K, (**b**) 100 K, (**c**) 230 K, and (**d**) 300 K. Emission from the LPB and MPB dispersions, as well as the uncoupled QW excitons, are indicated. Simulated data for LPB, MPB, UPB and cavity mode are presented as solid lines, while the corresponding exciton levels in the QWs and the GaN spacer, as dashed lines. Data obtained from membrane 1.

To establish the polaritonic properties of the full microcavity structures, a study of angle-resolved PL as a function of temperature is performed on a membrane, henceforth labelled “membrane 1”, using the k-space setup in imaging mode (*cf*. Methods). Figure 2a depicts the lower polariton branch (LPB) dispersion at 20 K, showing distinct strong coupling behaviour at large angles, where the LPB flattens out due to interaction of the cavity mode with the QW excitons. The position of the QW excitons is marked by the straight line corresponding to emission from “uncoupled” QW excitons, i.e. weakly-coupled QWs which are not at the antinodes of the standing wave. The upper polariton branch (UPB) is not observed in our experiments, something typical for nitride microcavities, where UPB features are either weak^12^ or not visible^11^.

As the temperature increases (*cf*. Fig. 2b–d), the QW exciton approaches the cavity mode and the LPB dispersion redshifts and becomes flatter. The LPB-to-exciton redshift ratio is around 2:3. At 230 K (Fig. 2c), we distinguish an additional branch at large angles with dispersive characteristics, which corresponds to the middle polariton branch (MPB) due to photon coupling with the excitons located at the 25 nm GaN spacer (*cf*. sample description in Methods). In Supplementary Fig. S3, the MPB is visible already at 80 K in the less negatively-detuned “membrane 2”. At 300 K (Fig. 2d), the uncoupled excitons lose intensity while the dispersive nature of the MPB and its anti-crossing with the LPB becomes more visible. An increase in the LPB linewidth is also observed with temperature, in line with phonon-related exciton broadening^27^.

The room temperature PL spectrum obtained at 10° from membrane 1, using the k-space setup in spectroscopy mode, is shown in Fig. 1c (solid line). The narrow line at 3.402 eV having a linewidth of 10.8 meV corresponds to the LPB, whereas the weaker peak at 3.453 eV is associated to uncoupled QW excitons. In order to extract the cavity Q-factor, a transfer matrix model^28, 29^ is used to estimate the absorption spectrum at an incident angle of 10°, which is then used to fit the PL data assuming direct proportionality between absorption and spontaneous emission^30^. Two excitons are defined in the model: the QW exciton at 3.453 eV and the GaN spacer exciton at 3.422 eV, each containing contributions from the A and B excitons of the respective sections, and having a homogeneous linewidth of 28 meV at 300 K. The latter was confirmed by PL measurements on bulk and QW samples, giving very similar results. Inhomogeneous broadening effects are ignored here, based on the much smaller exciton linewidths at low temperature. As shown in Fig. 1c, the model (red-dashed line) precisely simulates the experimental data assuming a cavity mode at 3.415 eV with linewidth of ≈1.93 meV, membrane thickness of ≈206 nm, and losses of 50 cm^−1^ and 100 cm^−1^ in the DBR mirrors and active region, respectively. The corresponding Q-factor is ≈1770, which is close to the theoretical Q-factor of ≈2350, estimated based on the same model, assuming no losses in the cavity.

To account for the observed polariton branches and estimate the Rabi splitting of the system, a Hamiltonian model^29^ described in detail in the Supplementary Information is used to simulate the angle-dependent data at 300 K, with parameters in line with the transfer matrix model. The two excitons, indicated as red dashed lines in Fig. 2d, interact with the cavity mode (green line) according to their respective coupling constants, forming three polariton branches and reproducing precisely the experimental dispersions. The unusual shape of the dispersions at the anticrossing between LPB and MPB is due to the relatively weak coupling constant of the bulk excitons in our structure, as discussed in the Supplementary Information. The splitting of the cavity mode and UPB at large angles is due to TE/TM splitting^31^. The Rabi splitting of the system is estimated to be ≈64 meV at the anti-crossing point between the LPB and UPB dispersions. To ensure the accuracy of the model, the PL response of two other membranes - membranes 2 and 3, with different detunings, is accurately simulated (*cf*. Supplementary Fig. S4) by simply varying their respective thickness, keeping all other parameters constant, including the coupling strength of the excitons, returning very similar Rabi splitting values.

**Figure 3:**
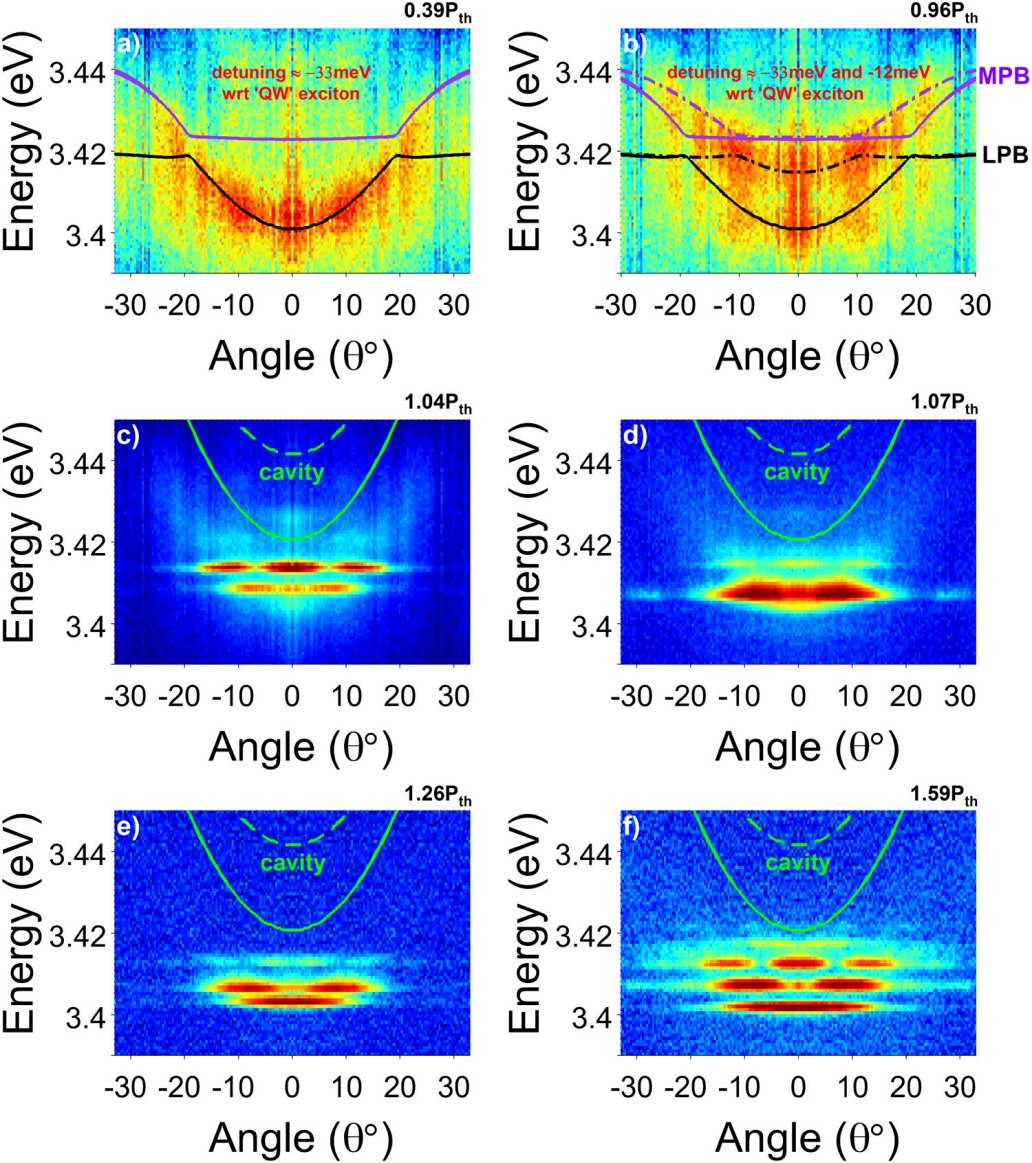
Angle-dependent room-temperature PL at various excitation powers around threshold. (**a**) 0.39 · P_th_, (**b**) 0.96 · P_th_, (**c**) 1.04 · P_th_, (**d**) 1.07 · P_th_, (**e**) 1.26 · P_th_, and (**f**) 1.59 · P_th_. The polariton condensation above threshold is associated to a distinct multimode k-space pattern, far below the theoretical cavity mode dispersions corresponding to LPB_1_ (solid line) and LPB_2_ (dashed line). Data obtained from membrane 5.

**Figure 4:**
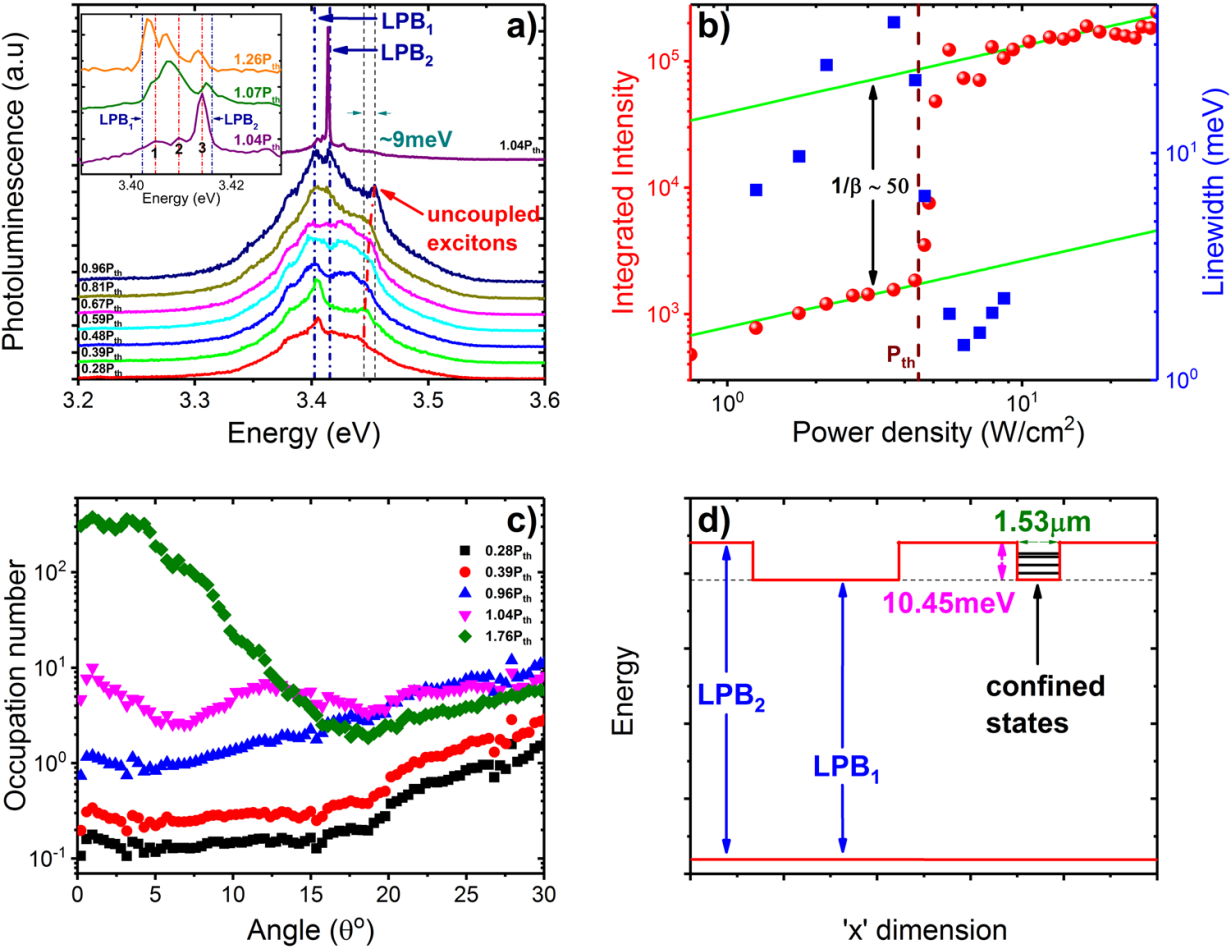
Polariton laser characteristics and sample schematic in line with observations. (**a**) PL spectra obtained from membrane 5 at 300 K using the k-space setup in spectroscopy mode, for power densities between 0.28 · P_th_ and 1.04 · P_th_. Inset: PL spectra above threshold, for power densities between 1.04 · P_th_ and 1.26 · P_th_, revealing three emission lines in the energy range between the LPB_1_ and LPB_2_. (**b**) Integrated PL intensity (red circles) in the region around the LPB_1_ and LPB_2_, and linewidth (blue squares) of the LPB_1_ peak (below threshold) and of level ‘1’ peak (above threshold), as a function of pump power density. (**c**) Occupation number as a function of pump power density, from 0.28 · P_th_ to 1.76 · P_th_. (**d**) An energy schematic of membrane 5, depicting the two regions corresponding to LPB_1_ and LPB_2_, and highlighting the square-like pocket trap.

The non-linear optical properties have been studied on membrane 5 at 300 K, exciting with a pulsed laser at 266 nm. Figure 3 presents the evolution of the angle-dependent PL emission as a function of the excitation power, reported as the ratio with respect to the average pumping power at threshold (P_th_ = 4.5 W/cm^2^). All images here are obtained using k-space setup in imaging mode. In parallel, Fig. 4a presents the corresponding PL spectra around *k*_||_ = 0, recorded using the same setup in spectroscopy mode. At low powers (0.39 · P_th_), a dispersion curve is observed in Fig. 3a, similar to Fig. 2d, containing LPB and MPB contributions, as fitted (solid lines) using the Hamiltonian model. We denote the lower polariton branch as LPB_1_, also indicated in the spectra of Fig. 4a. In Fig. 4a, with increasing power, LPB_1_ broadens without any visible change of its energy position. At 0.81 · P_th_, a second peak appears at higher energy (LPB_2_), which becomes clearly resolved just below threshold, at 0.96 · P_th_. The two vertical dot-dashed lines are guides to the eye showing the evolution of the two LPB’s with power. The LPB_2_ is also observable in the k-space data of Fig. 3b, showing a clear dispersive behaviour, which is also simulated in the Hamiltonian model (dot-dashed line) by solely adjusting the cavity thickness with respect to LPB_1_. Figure 4b plots the power-dependent evolution of the spectrally integrated intensity around the LPB lines. The onset of lasing is visible at extremely low average power densities of 4.5 W/cm^2^ (P_th_), marked with a dashed line. This value is four times lower compared to similar experiments on GaN QW microcavities^12^ using same excitation source. The integrated intensity shows an S-like behaviour with pump power, corresponding to 1/β ≈ 50, about two orders of magnitude lower compared to previous works using dielectric DBR only on one side^11^. It should be noted that in the context of a polariton laser the β-factor represents the fraction of polaritons spontaneously-emitted in the mode undergoing stimulated scattering^11^.

Above threshold, the spectra reveal three narrow peaks in the energy range between LPB_1_ and LPB_2_, as depicted in the inset of Fig. 4a, marked as ‘1’, ‘2’ and ‘3’. Level ‘3’ has the highest intensity at threshold, followed by ‘2’ and finally ‘1’. The corresponding image in Fig. 3c, at 1.04 · P_th_, shows a distinct multimode pattern in k-space corresponding to levels ‘2’ and ‘3’, whereas the emission from level ‘1’ is relatively weak. The system is in the strong coupling regime, as visible from the dispersion being flatter and well below the cavity modes corresponding to LPB_1_ and LPB_2_ (green solid and dashed lines), confirming polariton lasing vs. photonic lasing. The different levels follow an interesting power-dependent behaviour as seen in Fig. 3c–f and inset of Fig. 4a. Level ‘3’, first blueshifts and then redshifts with increasing pump power, whereas levels ‘1’ and ‘2’ redshift, with ‘2’ gaining maximum intensity already at 1.07 · P_th_. At higher powers, maximum lasing occurs from level ‘1’ followed by ‘2’ and ‘3’.

In Fig. 4c, we plot the occupation number as a function of angle for various pump powers, defined for a given power as the ratio of the spectrally-integrated PL intensity at each angle over the corresponding cavity fraction ${|{C}_{{k}_{\parallel }}|}^{2}(\theta )$ shown in Supplementary Fig. S5. The occupation number is normalized to unity, at *k*_||_ = 0, just below threshold (0.96 · P_th_). At powers below threshold, the occupation increases linearly with power, and exhibit higher occupation at large angles due to the bottleneck effect^32–34^. The evidence of bottleneck up to threshold powers indicates that the relaxation time of LPB polaritons remains longer than their lifetime at *k*_||_ = 0. This implies that polariton lasing/condensation occurs in the kinetic^35^ rather than the thermodynamic regime^10^. Just above threshold, the occupation at larger angles somewhat subsides, with a tremendous increase around *k*_||_ = 0, as expected for a polariton laser^10–12, 26^.

Polariton lasing is further confirmed by the calculation of the exciton densities involved in the process, based on the excitation level and a simple system of rate equations described in the Supplementary Information. The obtained threshold polariton density per laser pulse and per QW at *k*_||_ = 0 is *N*_1_ ≈ 2.24 × 10^10^ cm^−2^. Considering the exciton fraction |*X*_0_|^2^ for LPB_1_ at *k*_||_ = 0 (*cf*. Supplementary Information and Supplementary Fig. S5a), the corresponding threshold exciton density at *k*_||_ = 0 is given by *N*_*X*_ = *N* · |*X*_0_|^2^ ≈ 7 × 10^9^ cm^−2^, which is 2.5 orders of magnitude lower than the exciton saturation density in GaN QWs^36^, and 3.5 orders of magnitude lower than the electron-hole pair density needed for population inversion in a standard GaN laser diode^37^. In line with the low exciton densities in our system, we also note the absence of any noticeable blueshift of LPB_1_ up to threshold (*cf*. Fig. 4a), in contrast to previous works where significant blueshifts have been reported^11–13^.

In fact, assuming that the blueshift of the LPB line is less than 0.2 meV, which is the detection limit of our system, we obtain as described in the Supplementary Information the upper bound for the total exciton density at threshold of *N*_2*D*_ ≤ 4.7 × 10^10^ cm^−2^. This value is more than 40 times smaller than the exciton saturation density in these QWs, further demonstrating polariton lasing.

**Figure 5:**
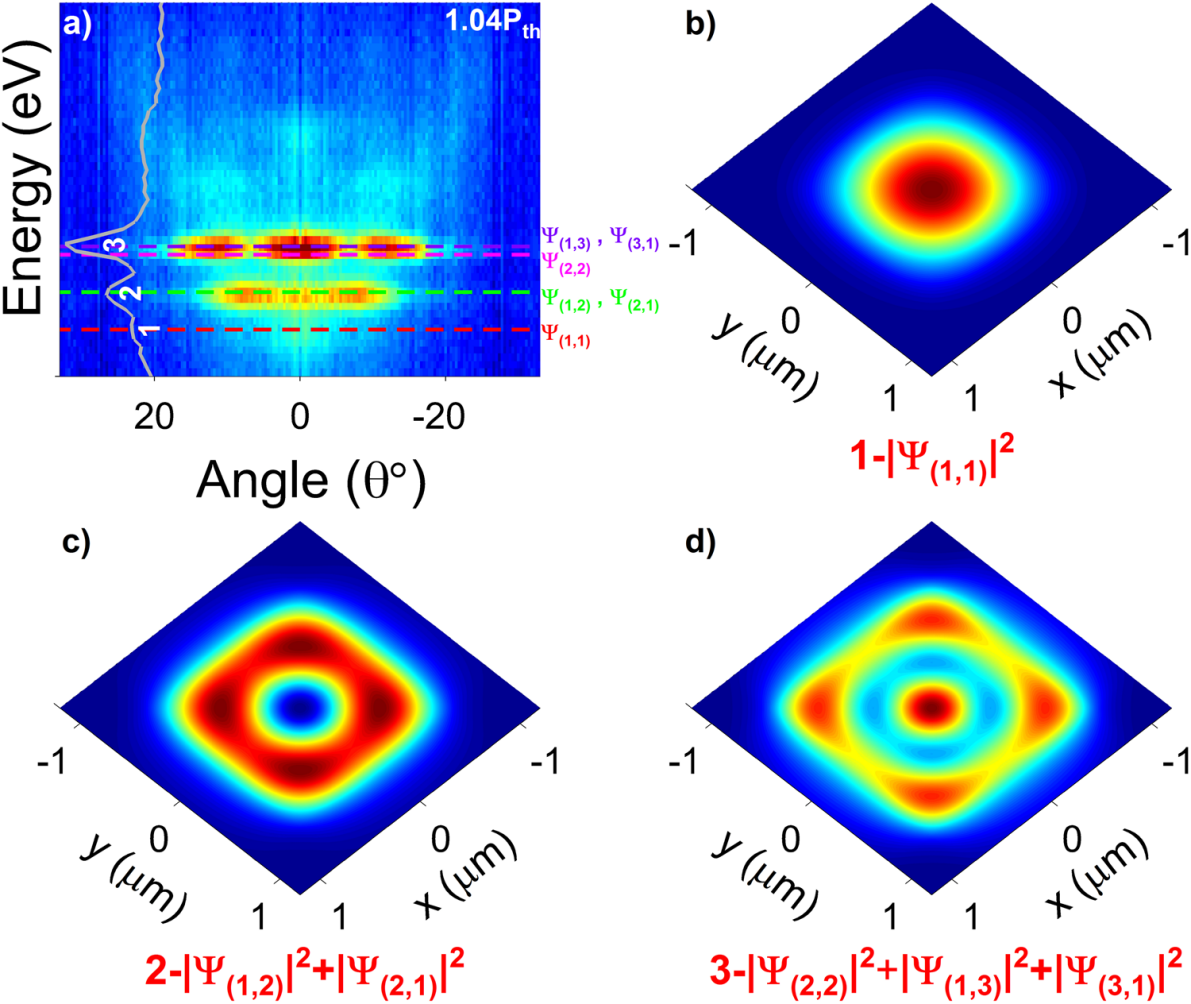
Quantum-confined polariton levels accounting for the observed k-space pattern. (**a**) Angle-dependent room-temperature PL from membrane 5, measured with an excitation power density of 1.04 · P_th_. The corresponding spectral data at *k*_||_ = 0° are plotted within the image, marking the levels ‘1’, ‘2’ and ‘3’. The dashed lines mark the respective simulated polariton levels. (**b**–**d**) Plots of the real-space squared wave-functions associated to (**b**) level ‘1’, (**c**) level ‘2’, and (**d**) level ‘3’.

Having established ultra-low-threshold polariton lasing, we turn now our attention to the k-space pattern of Fig. 3. The ordered pattern can be attributed to polariton condensation in a *single* spontaneously-formed zero-dimensional polariton trap^16, 38^, similarly to observations in engineered polariton landscapes^39–44^. Inhomogeneities related to the epitaxial growth and etching process can lead to membrane thickness variations of ≈1−2% (≈2−4 nm), which can act as polariton localization sites. Generally, the simultaneous observation of multiple confinement sites gives rise to a disordered pattern in k-space, like the one shown in Supplementary Fig. S6. To explain the data in Fig. 3, we assume the existence in our membrane of two types of uniformly-etched flat regions having a thickness difference of 3.6 nm, corresponding to the 20.9 meV energy difference between the cavity modes of the two LPBs in Fig. 3(c–f). The confinement site consists of a small-in-size LPB_1_ insertion, in a wider LPB_2_ region, as schematically depicted in the “inverted” energy profile of Fig. 4d. For simplicity, we assume a square-like trap, and solve the two-dimensional Schrödinger’s equation:

1 $$[-\frac{{\hbar}^{2}}{2{m}_{pol}}{\nabla }^{2}+V(x,y)]\,{{\Psi }}_{pol}(x,y)=E{{\Psi }}_{pol}(x,y)$$

where *V*(*x*, *y*) = *V*(*x*) + *V*(*y*) is the in-plane square-shaped potential trap having a depth of ~20.9 meV, *Ψ*_*pol*_ (*x*, *y*) is the polariton envelope wavefunction and *m*_*pol*_ is the polariton effective mass, estimated here to be 6.87 × 10^−5^ times the free electron mass based on the cavity/exciton fraction for LPB_1_ at *k*_||_ = 0. Using the trap width as the sole adjustable parameter, the discrete (1, 2, 3) energy levels in Fig. 5a just above threshold (1.04 · P_th_), can be nicely reproduced by the simulations (dashed lines) for a trap width of 1.53 μm. The real-space wavefunctions fitting the three levels (1, 2, 3) are plotted in Fig. 5(b–d). Using the standard notation for two-dimensional wavefunctions in a square potential well, the ground wavefunction *Ψ*_*pol*_ (1, 1) shown in Fig. 5b, accurately simulates level ‘1’, both in energy and shape in k-space. Similarly, the superposition of the degenerate states *Ψ*_*pol*_ (1, 2) and *Ψ*_*pol*_ (2, 1), shown in Fig. 5c, precisely matches the energy location of level ‘2’, as well as its distribution in k-space with a central node. Finally, level ‘3’ is located between the levels associated to *Ψ*_*pol*_ (2, 2) and the degenerate states *Ψ*_*pol*_ (1, 3) and *Ψ*_*pol*_ (3, 1). The superposition of these three states in Fig. 5d reproduces the k-space pattern of level ‘3’.

The variation in the relative intensity of levels 1, 2, and 3 with the pump power, displayed in Fig. 3c–f and inset of Fig. 4a, can be understood as follows. At low power, most of the polariton emission comes from the LPB_1_. Polaritons corresponding to the LPB_2_ relax either into the LPB_1_ regions or into the square-like trap, losing visibility at low powers. With increasing pump power, the polariton density increases, producing a broadening of LPB_1_. At powers just below threshold, the occupation of LPB_1_ seems to saturate, leading to a significant increase of the polariton density in the LPB_2_. Interestingly, polariton lasing occurs first at the confined levels of the trap, strongly suggesting the beneficial role of zero-dimensional confinement in the condensation process^45^, most likely due to relaxation of the exciton-phonon selection rules. Just above threshold, at 1.04 · P_th_, level ‘3’ has the highest intensity, followed by level ‘2’ and ‘1’. This can be explained by the longer polariton lifetime of level ‘3’, as it is closer to the excitonic reservoir than the lower levels. In other words, it is easier for the polariton population to build-up in level ‘3’, followed by level ‘2’ and finally ‘1’. The high intensity of level ‘3’ just above threshold, can also be attributed to the fact that it consists of three near-degenerate states, in comparison with level ‘2’ consisting of two degenerate states, and finally level ‘1’ having just a single state^38^. Beyond threshold, stimulated scattering^33, 34^ takes over, driving more efficiently polaritons into the lower states, as shown in the inset of Fig. 4a. Therefore at 1.26 · P_th_, level ‘1’ has the highest intensity, followed by levels ‘2’ and ‘3’. It should be noted that the power dependence of the multi-level emission above threshold is consistent with the fact that our polariton laser is still in the kinetic regime.

To conclude, an ultra-low threshold GaN polariton laser is demonstrated at room temperature, using a high-Q, all-dielectric, quantum-well microcavity, with a spontaneously-formed zero-dimensional trap. Polariton lasing is observed at carrier densities, 2.5 orders of magnitude lower than the exciton saturation density and 3.5 orders of magnitude lower than the electron-hole pair densities needed for population inversion in a conventional laser. The lasing spectra reveal an ordered pattern in k-space, attributed to polariton condensation at discrete levels of a single confinement site. The zero-dimensional confinement along with the superior material and optical quality of the structure, explain the enhanced characteristics of our polariton laser. Our results are likely to spur new activity in the area of robust room temperature polaritonics.

## Methods

The GaN layers are grown along the [0001] direction by plasma-assisted molecular beam epitaxy (MBE), on an n-type GaN template grown on c-sapphire by metalorganic chemical vapour deposition. The basic structure, depicted in Fig. 1a, consists of 33 pairs of GaN/Al_0.05_Ga_0.95_N (2.7 nm/2.7 nm) QWs deposited on a 25-nm-thick GaN spacer layer, which separates the QWs from the 25-nm-thick In_0.12_Ga_0.88_N sacrificial layer. The room-temperature PL emission of the In_0.12_Ga_0.88_N layer peaks at 430 nm. The GaN/Al_0.05_Ga_0.95_N heterostructure is not as “shallow” as one may think based on the low Al-content. The bandgap difference between well and barrier layers is already ~100 meV, which including strain effects is distributed 60:40 in the conduction and heavy-hole lineups, respectively, providing a good base for sufficient confinement. In addition, the carrier confinement is further enhanced by the sawtooth-like potential profiles due to the polarization-induced internal fields, present in all nitride heterostructures grown along a polar axis. Experimentally, we know that the thermal escape over the barrier layers is not a limiting factor in our case based on the relatively small PL intensity drop between low and room temperature, and the fact that the corresponding activation energy deduced from the Arrhenius plot of Supplementary Figure S2a is ~26 meV, which is three times smaller compared to the ~75 meV energy difference between QW emission and barrier energy gap. The dimensions of the membranes are defined by means of optical lithography using a thick photoresist AZ 9260. Then, samples are dry etched in a conventional Reactive Ion Etching system using BCl_3_ and Cl_2_, leading to rectangular mesas 700 nm deep. The remaining photoresist mask is removed by acetone/propanol/Di-H_2_O, while a short exposure to O_2_ plasma further ensures that no photoresist residues are left on the sample surface. Then, PEC etching of the sacrificial layer is performed inside an electrochemical cell, using KOH electrolyte with a molarity as low as 0.0004, while illuminating the sample with a 405 nm laser diode. The detailed description of the procedure can be found elsewhere^19, 21^. An optical microscope image of the sample after PEC etching (Supplementary Fig. S1a) shows detached membranes lying on the surface. For the transfer process, a proposed liquid-mediated adhesion mechanism is employed in order for the membranes to be detached from the top of the mesas and transfer top-face down onto a DBR structure, deposited on sapphire by e-beam evaporation and consisting of 11 pairs of λ/4 SiO_2_/Ta_2_O_5_ layers with respective nominal thicknesses of 59.6 nm and 44 nm, forming thus a half-microcavity sample (inset of Supplementary Fig. S1c). The full microcavity is completed by covering the membrane with 10 pairs of λ/4 HfO_2_/Al_2_O_3_ (40.3 nm/50.6 nm) DBRs using atomic layer deposition (ALD). The cross-sectional scanning electron microscopy (SEM) image in Fig. 1b shows a membrane sandwiched between the two sets of DBRs.

All the results in this paper are based on five specific membranes, which are described in Table 1. Their precise thickness is estimated from linear transfer matrix and Hamiltonian model simulations. It should be noted that in these calculations, the membrane thickness is the only adjustable parameter.

**Table 1:** Description of the membranes used in this study.

Name	Membrane thickness	Type of measurement
Membrane 1	≈206 nm	All basic optical characterization, including temperature dependence and estimation of Rabi splitting
Membrane 2	≈200 nm	Confirmation of MPB dispersion at lower temperatures
Membrane 3	≈201 nm	Confirmation of the determined Rabi splitting
Membrane 4	≈217 nm	Confirmation of the determined Rabi splitting
Membrane 5	Two regions with varying thickness of 205 nm and 202 nm.	Nonlinear and polariton lasing measurements

Most optical measurements were performed using an Acton triple-grating imaging spectrograph, with a focal length of 0.5 m, a resolution of 0.02 nm at 435.8 nm, and an accuracy of ±0.02 nm. The spectra were recorded using a 600 grooves/mm grating, blazed at 300 nm. For PL measurements, the sample is excited with a continuous wave He-Cd laser emitting at 325 nm. The lasing measurements are performed by excitation with a frequency-quadrupled Nd-YAG (neodymium-doped yttrium aluminum garnet) laser emitting at 266 nm, with a pulse repetition rate of 7.58 kHz and a pulse width of 0.51 ns. At this wavelength, the absorption coefficient of GaN is ~1.8 · 10^5^ cm^−1^ ^46^, implying that more than 97% of the incident pump light is absorbed by the 200 nm-thick membranes during the lasing experiments. Reflectivity characterization is performed using a 150 W Xe lamp. For PL and reflectivity measurements, samples are placed on the cold finger of a He closed-circuit cryostat. The excitation beam, at normal incidence, is focused using an aspherical lens with a numerical aperture NA = 0.63, and the PL/reflectivity signal is collected through the same optical path using a beam splitter. The focus of the aspherical lens is adjusted for the main emission wavelength, around 360 nm. As a result, the 325 nm laser is focused down to a diameter of 9 μm, and the 266 nm laser presents a spot size of 60 × 10 μm^2^, due to its elliptical profile. The PL/reflectivity is focused onto the spectrometer using a final collection lens.

For angle-dependent measurements, an additional lens (Fourier plane imaging lens) is positioned before the final lens, allowing PL/reflectivity imaging of the Fourier plane into the spectrometer (k-space setup). This k-space setup can be operated in two modes: (1) spectroscopy mode, where signal from only a few angles is probed and averaged, giving a single spectrum, and (2) imaging mode, where the entire k-space as a function of angle, limited by the NA of the system, is recorded in a single shot.

Time-resolved PL measurements were performed using a Hamamatsu M5675 Streak camera and a frequency-tripled Ti:Sapphire laser emitting at 266 nm, with a pulse width of 150 fs, repetition rate of 76 MHz and an average power density of 2 W/cm^2^.

## Electronic supplementary material

Supplementary Information
