# Supplementary material for: Ultra-low threshold polariton lasing at room temperature in a GaN membrane microcavity with a zero-dimensional trap
Source: Sci Rep. 2017 Jul 17;7:5542. doi: 10.1038/s41598-017-06125-y (PMC5514101; doi:10.1038/s41598-017-06125-y)
Supplement: Supplementary file 1 — Supplementary Information [file 41598_2017_6125_MOESM1_ESM.pdf]

# Ultra-low threshold polariton lasing at room temperature in a GaN membrane microcavity with a zero-dimensional trap

R. Jayaprakash[1,2], F. G. Kalaitzakis[1,2], G. Christmann[2], K. Tsagaraki[2], M. Hocevar[3,4], B. Gayral[3,5], E. Monroy[3,5], and N. T. Pelekanos[1,2]

[1]Department of Materials Science and Technology, University of Crete, P.O. Box 2208, 71003 Heraklion, Greece

[2]Microelectronics Research Group, IESL-FORTH, P.O. Box 1385, 71110 Heraklion, Greece

[3]Université Grenoble-Alpes, 38000 Grenoble, France

[4]CNRS, Inst NEEL, F-38000 Grenoble, France

[5]CEA, INAC-PHELIQS, 17 rue des Martyrs, 38000 Grenoble, France

# SUPPLEMENTARY INFORMATION

## Threshold exciton densities

Polariton lasing can be confirmed by estimating the threshold exciton density per QW, per laser pulse ($N_{2D}$). The value of $N_{2D}$ is often estimated from the blueshift of the LPB line at $k_{\parallel}$ = 0 when increasing the pump power up to the threshold value ($\Delta E_{LPB}$), using the following relation[1,2]:

$$\Delta E_{LPB} = V_0 \cdot N_{2D} \qquad (1)$$

where $V_0$ is the matrix element of polariton-polariton interaction given by $V_0 = 6|X_0|^2 E_x^B {a_B^*}^2$, with $|X_0|^2$ being the exciton fraction at $k_{\parallel}$ = 0, $E_x^B$ the exciton binding energy, and $a_B^*$ the exciton Bohr radius. In our case, however, the blueshift is hardly observable (see Fig. 4a), which is consistent with the much lower exciton densities in our system compared to previous works. Instead, threshold carrier densities can be estimated based on a simple system of rate equations, coupling the LPB polariton densities at $k_{\parallel}$ = 0 and at the high-$k_{\parallel}$ "reservoir" states. Starting from the latter, the variation of the threshold polariton density in the reservoir per coupled QW and per laser pulse ($N_2$) can be written as

$$\frac{dN_2}{dt} = G - \frac{N_2}{\tau_2} - \frac{N_2}{\tau_{rel}} \qquad (2)$$

where $G$ is the carrier generation rate per QW per laser pulse at threshold, $\tau_{rel}$ the relaxation time of polaritons to the bottom of LPB, and $\tau_2$ the lifetime of polaritons in the reservoir states, which can be approximated by the excitonic lifetime $\tau_{exc}$ = 275 ps [*cf.* inset of Supplementary Fig. S1b]. Taking into consideration that $\tau_{rel}$ << $\tau_2$, at steady state, equation (2) gives:

$$G = \frac{N_2}{\tau_{rel}} \qquad (3)$$

Similarly, the variation of the threshold polariton density at $k_{\parallel}$ = 0 per coupled QW per laser pulse ($N_1$), can be written as:

$$\frac{dN_1}{dt} = \frac{N_2}{\tau_{rel}} - \frac{N_1}{\tau_1} \qquad (4)$$

where $\tau_1$ is the lifetime of polaritons at $k_{\|} = 0$. Taking the steady-state limit of equation (4), and using equation (3), we obtain the relation:

$$N_1 = G \cdot \tau_1 \qquad (5)$$

$G$ can be estimated from the threshold power density of ≈ 4.5 W/cm$^2$, which is the lowest ever reported value for a two-dimensional GaN-based microcavity, taking into account the 5% loss in the top DBR mirror determined by transmission experiments and making the following simplifying assumptions: (i) the incident light is fully absorbed by the membrane, (ii) we ignore the absorption of the GaN spacer layer and (iii) the generated carriers are equally distributed among the QW's. It should be noted that assumptions (i) and (ii) lead to an upper estimate of the threshold densities. The value of $\tau_1$ at $k_{\|} = 0$ is estimated from[3]:

$$\frac{1}{\tau_1} = \frac{|C_0|^2}{\tau_{cav}} + \frac{|X_0|^2}{\tau_{exc}} \qquad (6)$$

where $|C_0|^2$ and $|X_0|^2$ are the cavity and exciton fractions of $LPB_1$ at $k_{\|} = 0$, $\tau_{exc}$ = 275 ps, and $\tau_{cav}$ ≈ 0.34 ps is the cavity lifetime calculated from the Q-factor corresponding to the simulation of Fig. 1e. Since we ignore the absorption in the GaN spacer layer, the overall excitonic fraction can be taken as $|X_0|^2 = |X_0^{QW}|^2 + |X_0^{GaN}|^2$ (*cf.* Supplementary Fig. S4a), which has been independently verified using a two-level coupled oscillator model[3]. Inserting these values into equation (6), we obtain $\tau_1$ = 0.498 ps. Introducing this value in equation (5), we obtain an upper estimate for $N_1$ of 2.24×10$^{10}$ cm$^{-2}$. The corresponding exciton density ($N_X$) at $k_{\|}$ = 0 can be obtained considering the exciton fraction ($|X_0|^2$) of $LPB_1$ at $k_{\|} = 0$, through the relation:

$$N_X = N_1 \cdot |X_0|^2 \qquad (7)$$

Substituting for $N_1$ and $|X_0|^2$, $N_X$ is estimated to be ≈ 7×10$^9$ cm$^{-2}$, which is 2.5 orders of magnitude below the exciton saturation density $n_{sat}$ ≈ 2×10$^{12}$ cm$^{-2}$.[4] The exciton saturation density is also independently estimated as described in references[4,5], using a QW exciton binding energy $E_x^B$ = 30 meV[4,6] and a Bohr radius $a_B^*$ = 2.7 nm. Since at threshold, most of the lasing comes from level '3', as opposed to level '1' (see inset of Fig. 4a), an estimate of $N_1$ is also made for $LPB_2$, which turns out to be very similar to the above estimated value, in spite of the smaller detuning.

The above threshold carrier densities at $k_{\|} = 0$ are consistent with the absence of a measurable blueshift of the LPB line. In fact, assuming that $\Delta E_{LPB}$ in equation (1) is smaller than the detection limit of our system of 0.2 meV and using $E_x^B = 30$ meV, $a_B^* = 2.7$ nm and $|X_0|^2 = 0.32$, we can obtain an upper bound for $N_{2D} \leq 4.7\times10^{10}$ cm$^{-2}$, which is more than 40 times lower than the exciton saturation density in these QWs further confirming polariton lasing.

To further check the validity of our estimates, the exciton density in the "uncoupled" QWs ($N_{2D}^{uncoupled}$), per laser pulse and per QW, can be obtained from the following equation:

$$\frac{dN_{2D}^{uncoupled}}{dt} = G - \frac{N_{2D}^{uncoupled}}{\tau_{exc}} \qquad (8)$$

$G$ and $\tau_{exc}$ are known, as per the discussion above, giving a steady-state estimate for the $N_{2D}^{uncoupled}$ of approximately $10^{12}$ cm$^{-2}$. Alternatively, $N_{2D}^{uncoupled}$ can be estimated from the blueshift of the PL emission from uncoupled QW excitons, when increasing the pump power up to threshold, which is $\Delta E_{exc} \approx 9$ meV from data in Fig. 4a. Slightly modifying equation (1), $\Delta E_{exc}$ can be written as

$$\Delta E_{exc} = V_{exc} \cdot N_{2D}^{uncoupled} \qquad (9)$$

where $V_{exc}$ is the matrix element of exciton-exciton interaction given by $V_{exc} = 6E_x^B {a_B^*}^2$. From this equation, we obtain $N_{2D}^{uncoupled} = 7 \times 10^{11}\ cm^{-2}$, which is very close to the value derived from the rate equation model.

## <u>3 x 3 coupled oscillator model</u>

The Hamiltonian ($H$) defining a 3 x 3 coupled oscillator model can be written as follows:

$$H = \begin{pmatrix} E_1(k_{\|}) - i\gamma_1 & 0 & g_1 \\ 0 & E_2(k_{\|}) - i\gamma_2 & g_2 \\ g_1 & g_2 & E_{cav}(k_{\|}) - i\gamma_{cav} \end{pmatrix} \qquad (10)$$

where $E_1(k_{\|})$, $E_2(k_{\|})$ and $E_{cav}(k_{\|})$ are the energies of the first exciton, second exciton and the cavity mode respectively, as a function of in-plane wave vector ($k_{\|}$). $\gamma_1$, $\gamma_2$ and $\gamma_{cav}$ are the corresponding linewidths of the first exciton, second exciton and the cavity mode. $g_1$ and $g_2$ are

the respective coupling constant's of the first and second excitons. The coupling constant $(g)$ of an exciton, is given by the following relation:

$$g = \hbar\sqrt{\frac{2\Gamma_0 c N_{QW}}{n_{cav} d^*_{cav}}} \qquad (11)$$

where $c$ is the velocity of light in air, $N_{QW}$ is the number of QW's, $n_{cav}$ is the effective refractive index of the cavity layer, $d^*_{cav}$ is the effective cavity length including the penetration into the mirrors and finally $\hbar\Gamma_0$ is given by:

$$\hbar\Gamma_0 = \frac{\pi e^2 \hbar}{4\pi\varepsilon_0 n_{cav} m c} \frac{f^{QW}_{osc}}{S} \qquad (12)$$

where $e$ is the charge of an electron, $\varepsilon_0$ is the vacuum dielectric constant, $m$ is the mass of an electron and $\frac{f^{QW}_{osc}}{S}$ is the QW oscillator strength per unit area. It should be noted that the expression for coupling constant $(g)$ is an approximate formula, derived under the following assumptions: (1) the cavity layer has a higher refractive index as opposed to its DBR constituents and (2) the QW's are placed exactly at the antinodes of the field distribution within the cavity layer, estimated for a resonant cavity photon energy $(E_{cav}(0))$.

The Hamiltonian $(H)$ of equation (10), can be diagonalised to determine the eigen-energies, giving $E_{LPB}(k_\parallel)$, $E_{MPB}(k_\parallel)$ and $E_{UPB}(k_\parallel)$, corresponding to the eigen-states of the coupled excitons and photons, where LPB, MPB and UPB denote the lower, middle and upper polariton branch. The subsequent normalised eigen-vectors for $LPB$, $MPB$ and $UPB$ are the following:

$$\begin{pmatrix} -X^1_{k_\parallel} \\ -X^2_{k_\parallel} \\ C_{k_\parallel} \end{pmatrix}_{LPB} \begin{pmatrix} X^1_{k_\parallel} \\ -X^2_{k_\parallel} \\ C_{k_\parallel} \end{pmatrix}_{MPB} \begin{pmatrix} X^1_{k_\parallel} \\ X^2_{k_\parallel} \\ C_{k_\parallel} \end{pmatrix}_{UPB} \qquad (13)$$

where $X^1_{k_\parallel}$, $X^2_{k_\parallel}$ and $C_{k_\parallel}$ are the Hopfield coefficients. $|X^1_{k_\parallel}|^2$, $|X^2_{k_\parallel}|^2$ are the exciton fractions corresponding to the two excitons and $|C_{k_\parallel}|^2$ represents the photon fraction, for the respective polariton branches. The Hopfield coefficients satisfy the following relation:

$$|X^1_{k_\parallel}|^2 + |X^2_{k_\parallel}|^2 + |C_{k_\parallel}|^2 = 1 \qquad (14)$$

The energy splitting ($\hbar\Omega_{VRS}$) between the respective branches at the anti-crossing point ($k_{\parallel}$ at which the cavity mode energy coincides with the exciton energy), can be referred to as the vacuum field Rabi splitting. It should be noted that, two Rabi splitting's can be observed in a 3 x 3 system: 1) between the LPB and MPB and 2) between the MPB and UPB. Further discussion about the model can be found in the following references[3,7].

## Comment on the unusual kink between LPB and MPB

The unusual kink at the anticrossing between LPB and MPB dispersions, which can be noticed in the fittings of Figures 2(d), 3(a) and 3(b), is a generic effect that occurs whenever the coupling constant decreases to values just above the transition between weak and strong coupling regimes. In the 3-level fittings of our system, containing both QW and bulk GaN excitons, the respective coupling constants are 0.031575 eV and 0.01005 eV. In other words, the coupling constant of the bulk GaN excitons is much weaker compared to the QW excitons, in line with the relatively weak Rabi splittings between LPB and MPB.

In order to make our point clear, in a simple way, we show in the Supplementary Figure S7, the outcome of 2-level calculations, where we only vary the coupling constant, keeping all other parameters intact (e.g. detunings). For relatively large coupling constants, we observe the typical "smooth" anticrossing behaviour, but as we decrease the coupling constant to the limit close to the weak coupling case, we observe a kink-like behaviour, similar to the one observed between LPB and MPB in the 3-level fittings of the manuscript.

## Refractive indices

The refractive indices used in the transfer matrix and coupled oscillator model is tabulated below:

| Material | Methodology | Reference |
|---|---|---|
| GaN | Sellmeier equation | [8] |
| AlGaN | bandgap dependent continuous | [9] |

| | | |
|---|---|---|
| | equation for nitride ternary alloys | |
| $SiO_2$ | Sellmeier equation | [10,11] |
| $Ta_2O_5$ | Sellmeier equation | [10,11] |
| $HfO_2$ | estimated from ellipsometry measurements, made on a 200nm thick, bare $HfO_2$ film on silicon | - |
| $Al_2O_3$ | estimated from ellipsometry measurements, made on a 200nm thick, bare $Al_2O_3$ film on silicon | - |
| Sapphire (substrate) | Sellmeier equation | [12] |


1. Tassone, F. & Yamamoto, Y. Exciton-exciton scattering dynamics in a semiconductor microcavity and stimulated scattering into polaritons. *Phys. Rev. B* **59,** 10830–10842 (1999).

2. Ciuti, C., Schwendimann, P., Deveaud, B. & Quattropani, A. Theory of the angle-resonant polariton amplifier. *Phys. Rev. B* **62,** R4825–R4828 (2000).

3. Deng, H., Haug, H. & Yamamoto, Y. Exciton-polariton Bose-Einstein condensation. *Rev. Mod. Phys.* **82,** 1489–1537 (2010).

4. Rossbach, G. *et al.* High-temperature Mott transition in wide-band-gap semiconductor quantum wells. *Phys. Rev. B* **90,** 201308 (2014).

5. Christmann, G. *et al.* Large vacuum Rabi splitting in a multiple quantum well GaN-based microcavity in the strong-coupling regime. *Phys. Rev. B* **77,** 085310 (2008).

6. Shields, P. A., Nicholas, R. J., Grandjean, N. & Massies, J. Magnetophotoluminescence of GaN/AlGaN quantum wells: valence band re-ordering and excitonic binding energies. *Phys. Rev. B* **63,** 245319 (2001).

7. Christmann, G. III-nitride Based Microcavities: Towards Polariton Condensation at Room Temperature. (EPFL, 2009).

8. Barker, A. S. & Ilegems, M. Infrared Lattice Vibrations and Free-Electron Dispersion in GaN. *Phys. Rev. B* **7,** 743–750 (1973).

9. Laws, G. M., Larkins, E. C., Harrison, I., Molloy, C. & Somerford, D. Improved refractive index formulas for the Al[x]Ga[1−x]N and In[y]Ga[1−y]N alloys. *J. Appl. Phys.* **89,** 1108 (2001).

10. Weber, M. J. *Handbook of Optical Materials*. (CRC Press, Boca Raton, 2002).

11. Gao, L., Lemarchand, F. & Lequime, M. Exploitation of multiple incidences spectrometric measurements for thin film reverse engineering. *Opt. Express* **20,** 15734 (2012).

12. Kelly, R. L. Program of the 1972 Annual Meeting of the Optical Society of America. *JOSA* **62,** 1336 (1972).

<u>**Supplementary Figures**</u>

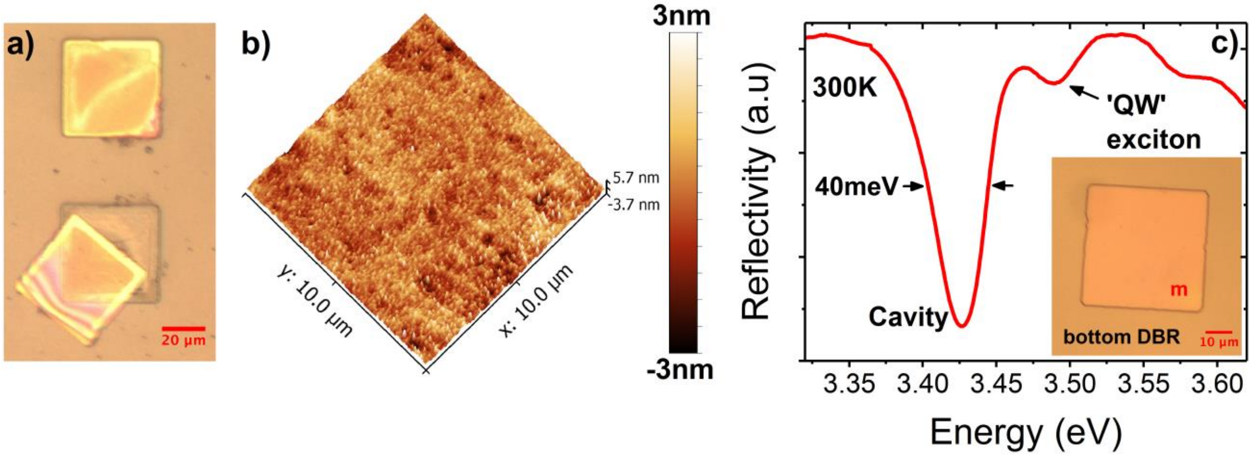


**Supplementary Figure S1.** (**a**) Optical microscope image of membranes lying on the sample surface after PEC lateral etching. (**b**) AFM image of the etched surface (RMS roughness ≈ 0.65 nm). (**c**) Reflectivity response at room temperature from a half-microcavity structure consisting of a membrane lying on a dielectric DBR mirror (optical microscope image in the inset). The QW exciton and the cavity mode, with a Q-factor ≈ 85, are identified.

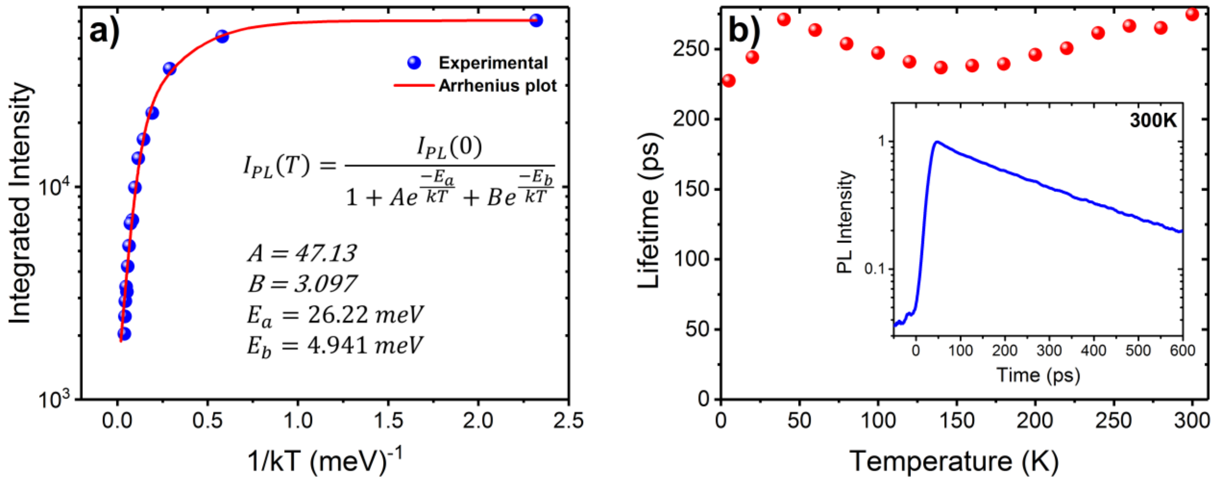


**Supplementary Figure S2.** (a) Arrhenius plot of the integrated PL intensity versus inverse temperature. The Arrhenius fit through the data points gives a main activation energy of ~26 meV. (b) PL decay time extracted as a function of temperature from the dominant QW peak, measured simultaneously in the time-resolved PL setup with an average excitation power density of about 2 W/cm$^2$. The inset of Figure (b) depicts the time-resolved PL decay curve at 300 K, with an exponential decay time of 275 ps.

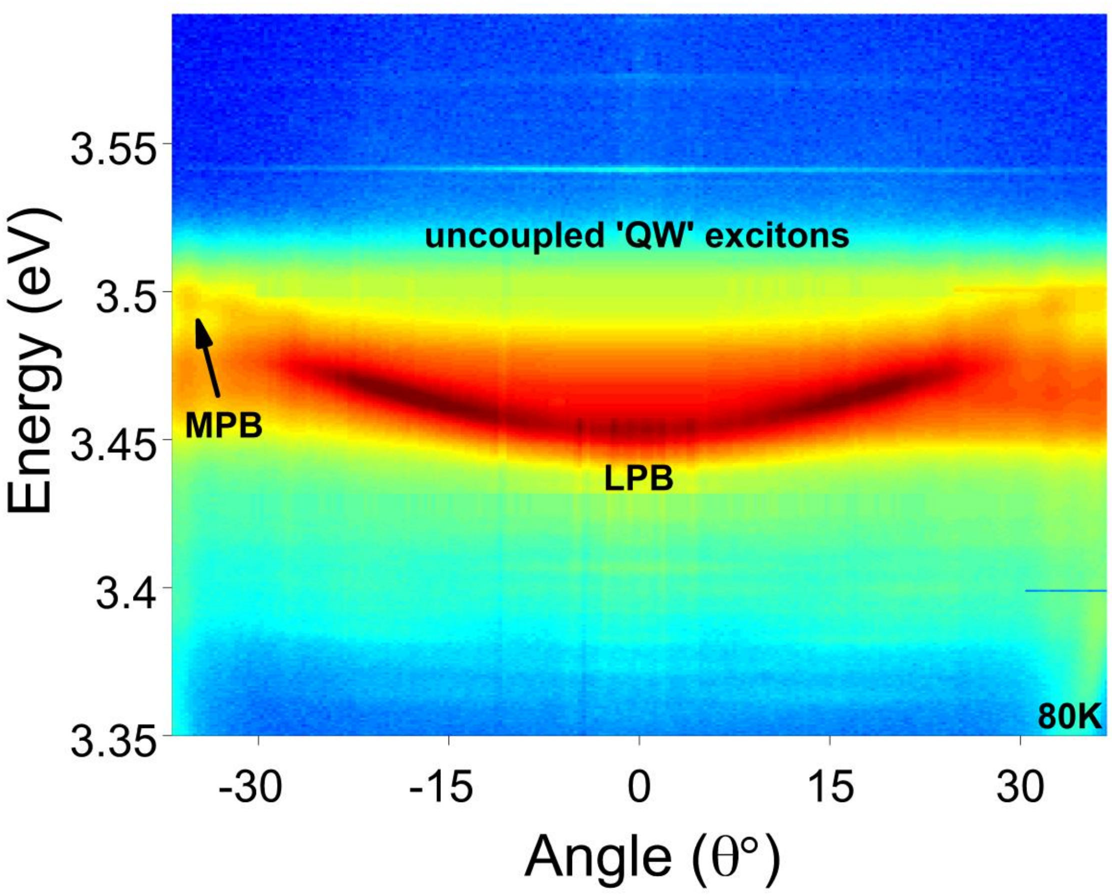


**Supplementary Figure S3.** Demonstration of strong coupling behaviour revealing the LPB and MPB dispersions at 80K, in the less negatively-detuned - membrane 2.

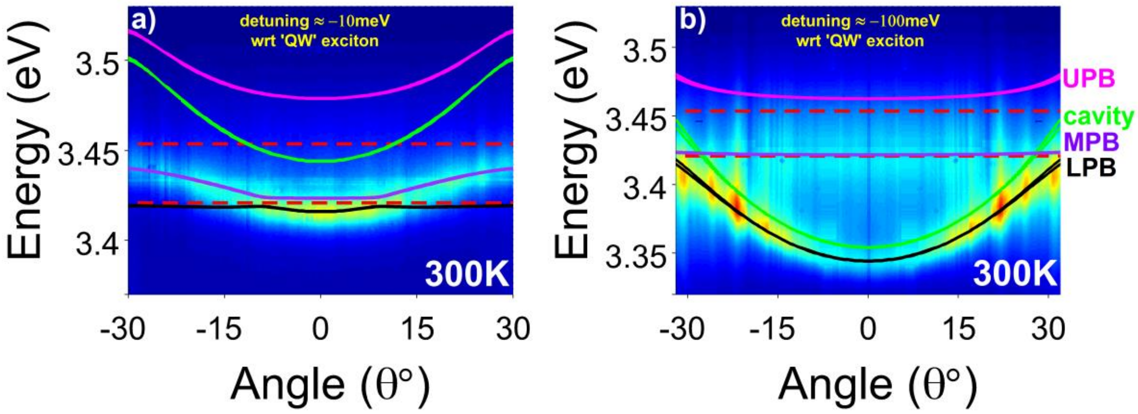


**Supplementary Figure S4.** Demonstration of strong coupling behaviour at 300 K, revealing the LPB and MPB dispersions, for (a) membrane 3, and (b) membrane 4, which are two membranes having different detuning. The solid and dashed lines correspond to simulation curves, using exactly the same colour code as in Fig. 2d. In the simulation, which precisely simulates the experimental data, the only adjustable parameter was the cavity thickness, while all other parameters, including the coupling strength of the excitons were left constant.

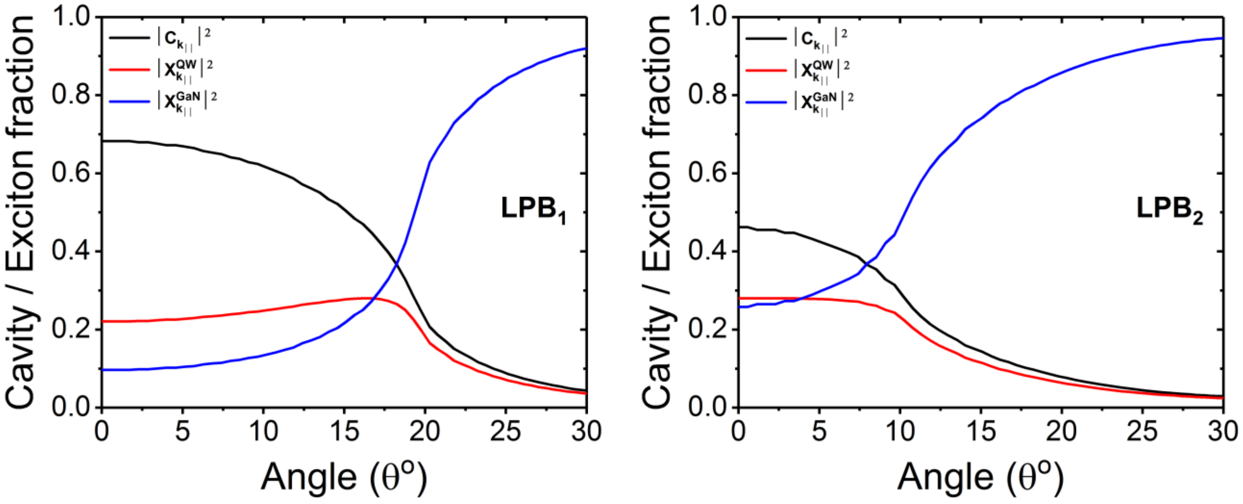


**Supplementary Figure S5.** The cavity / exciton fraction associated to: (a) $LPB_1$, and (b) $LPB_2$, calculated from Hopfield coefficients.

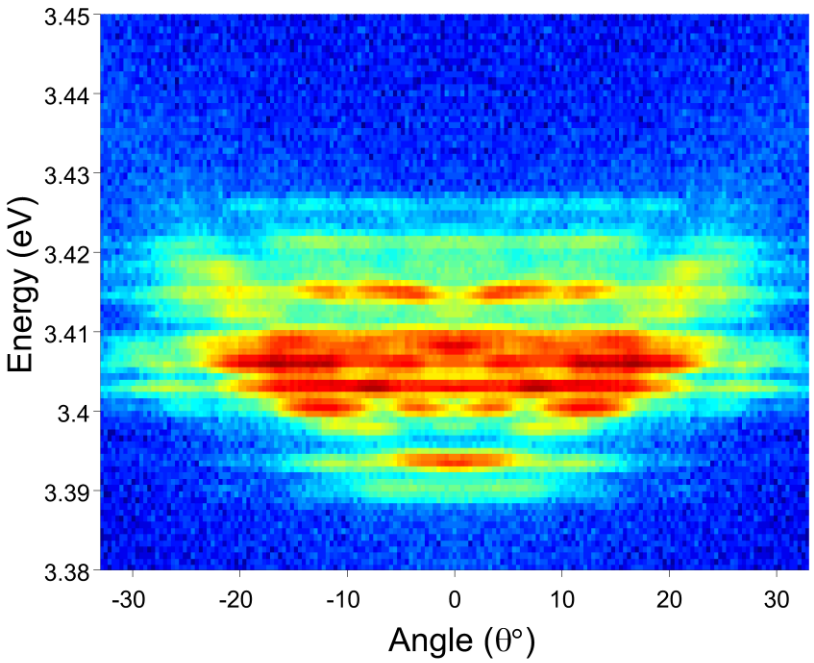


**Supplementary Figure S6.** Room-temperature angle-dependent PL emission above threshold obtained from a membrane having multiple confinement sites, giving rise to a disordered pattern.

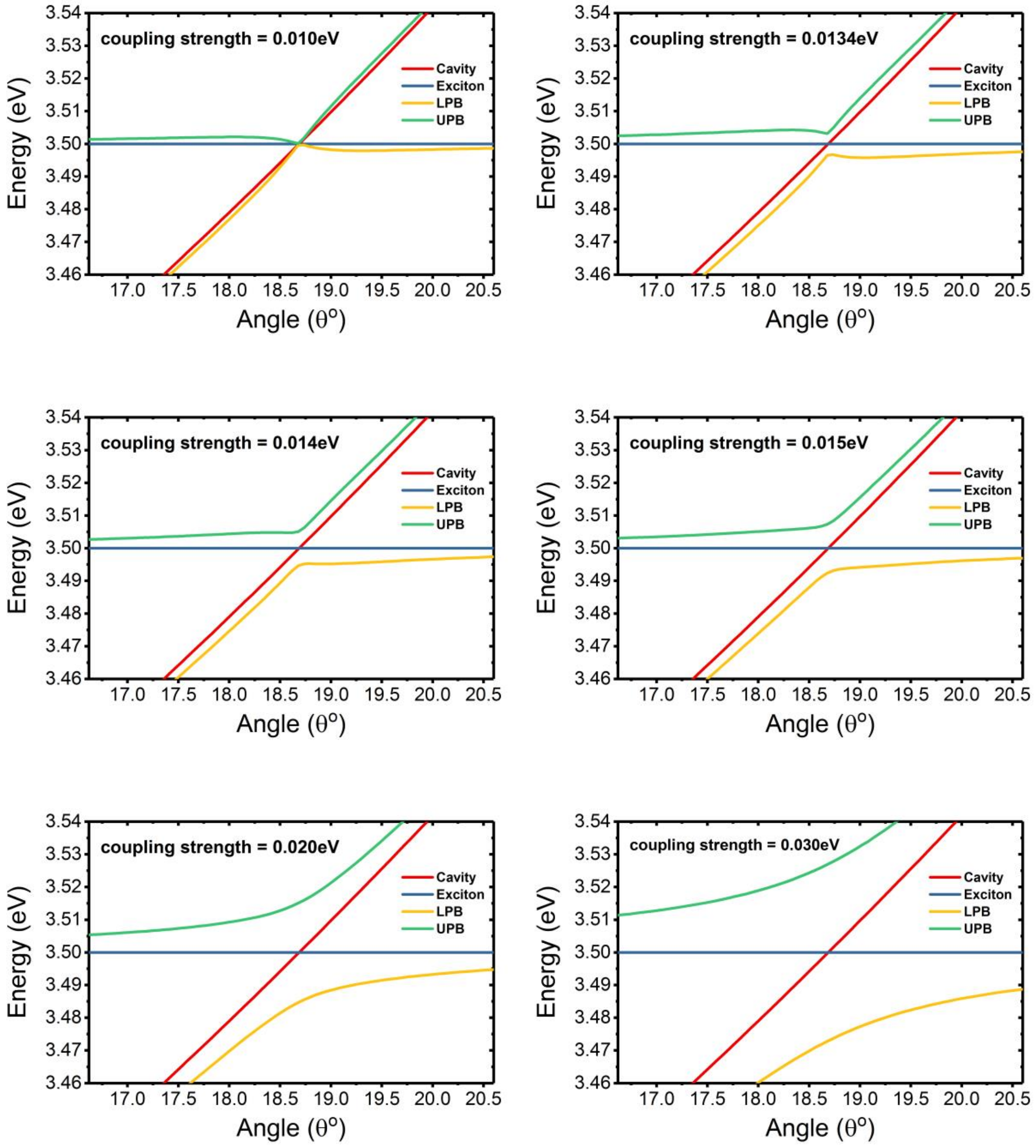


**Supplementary Figure S7.** The effect on the polariton branches as the coupling constant is tuned from the weak / intermediate to the strong coupling regime. The exciton and cavity linewidths are 0.028eV and 0.002eV respectively, the detuning is fixed at –0.3eV, and the coupling constant ($g$) is varied in the following manner: (a) 0.010eV (b) 0.0134eV (c) 0.014eV (d) 0.015eV (e) 0.020eV and (f) 0.030eV, where the first four are in the weak / intermediate coupling regime whereas the latter two are in the strong coupling regime.
